# Detrimental Effect of Various Preparations of the Human Amniotic Membrane Homogenate on the 2D and 3D Bladder Cancer *In vitro* Models

**DOI:** 10.3389/fbioe.2021.690358

**Published:** 2021-06-25

**Authors:** Aleksandar Janev, Taja Železnik Ramuta, Larisa Tratnjek, Žiga Sardoč, Hristina Obradović, Slavko Mojsilović, Milena Taskovska, Tomaž Smrkolj, Mateja Erdani Kreft

**Affiliations:** ^1^Institute of Cell Biology, Faculty of Medicine, University of Ljubljana, Ljubljana, Slovenia; ^2^Laboratory for Experimental Hematology and Stem Cells, Institute for Medical Research, University of Belgrade, Belgrade, Serbia; ^3^Department of Urology, University Medical Centre Ljubljana, Ljubljana, Slovenia; ^4^Chair of Surgery, Faculty of Medicine, University of Ljubljana, Ljubljana, Slovenia

**Keywords:** cancer, urothelium, 2D and 3D *in vitro* models, light and electron microscopy, proliferation, cell cycle

## Abstract

Despite being among the ten most common cancers with high recurrence rates worldwide, there have been no major breakthroughs in the standard treatment options for bladder cancer in recent years. The use of a human amniotic membrane (hAM) to treat cancer is one of the promising ideas that have emerged in recent years. This study aimed to investigate the anticancer activity of hAM homogenate on 2D and 3D cancer models. We evaluated the effects of hAM homogenates on the human muscle invasive bladder cancer urothelial (T24) cells, papillary cancer urothelial (RT4) cells and normal porcine urothelial (NPU) cells as well as on human mammary gland non-tumorigenic (MCF10a) cells and low-metastatic breast cancer (MCF7) cells. After 24 h, we observed a gradual detachment of cancerous cells from the culture surface, while the hAM homogenate did not affect the normal cells. The most pronounced effect hAM homogenate had on bladder cancer cells; however, the potency of their detachment was dependent on the treatment protocol and the preparation of hAM homogenate. We demonstrated that hAM homogenate significantly decreased the adhesion, growth, and proliferation of human bladder invasive and papillary cancer urothelial cells and did not affect normal urothelial cells even in 7-day treatment. By using light and electron microscopy we showed that hAM homogenate disrupted the architecture of 2D and 3D bladder cancer models. The information provided by our study highlights the detrimental effect of hAM homogenate on bladder cancer cells and strengthens the idea of the potential clinical application of hAM for bladder cancer treatment.

## Introduction

The incidence of bladder cancer is steadily increasing, especially in industrialized countries (Bray et al., 2018). The disease is more prevalent in men, who accounted for 440,864 out of 573,278 new cases diagnosed in 2020 (Sung et al., 2021). The predominant histologic type of bladder cancer is urothelial carcinoma and out of all newly diagnosed cases, approximately 75% are non-muscle invasive bladder cancer (NMIBC) and 25% are muscle-invasive bladder cancer (MIBC) (Sanli et al., 2017; Patel et al., 2020).

The main challenge of NMIBC management is its high recurrence rate. Namely, approximately 50–70% of NMIBC cases will recur and 15–20% will progress to MIBC (Sylvester et al., 2006; Isharwal and Konety, 2015; Knowles and Hurst, 2015; Patel et al., 2020). Due to the high recurrence rate and consequent disease monitoring requirements, bladder cancer has one of the highest lifetime treatment costs per patient among cancers (Sievert et al., 2009). The prognosis for patients with NMIBC is very encouraging as the 5-year survival rate is approximately 90%, however, the 5-year survival rate of patients with the metastatic disease is still merely 6% (Funt and Rosenberg, 2017).

While research of tumor biology resulted in major therapeutic advances in several other cancers, systemic therapy for bladder cancer is developing more slowly. In the field of intravesical therapy for NMIBC there have not been any major changes, despite global shortage of Bacillus Calmette-Guerin (BCG) immunotherapy, which is the gold standard for treatment of intermediate and high risk NMIBC (Pettenati and Ingersoll, 2018; Patel et al., 2020). Moreover, despite the increased use of neoadjuvant and adjuvant systemic chemotherapy for MIBC, the long-term survival rates remain unchanged (Advanced Bladder Cancer (ABC) Meta-analysis Collaboration, 2005a, b; Zehnder et al., 2013; Hermans et al., 2016). Nevertheless, it is encouraging that the initial results of systemic immunotherapy in regard to the disease progression and survival are promising (Powles et al., 2014, 2017; Rosenberg et al., 2016; Apolo et al., 2017; Balar et al., 2017; Bellmunt et al., 2017; Farina et al., 2017; Sharma et al., 2017; Vaughn et al., 2018; Fradet et al., 2019). Overall, there is a great need for the development of novel therapeutic approaches that would improve survival and decrease the recurrence, particularly for NMIBC.

The human amniotic membrane (hAM) is a placenta-derived biomaterial that has a long history of use in regenerative medicine (Malhotra and Jain, 2014; Silini et al., 2015; Shakouri-Motlagh et al., 2017). It is composed of a monolayer of human amniotic epithelial cells (hAEC), basal lamina, and hAM stroma, which is further divided into a compact layer, a layer of human amniotic mesenchymal stromal cells (hAMSC) and a spongy layer (Parolini et al., 2008; Lee et al., 2016). Besides having properties that are beneficial for use in tissue engineering and regenerative medicine, such as promotion of epithelization (Koizumi et al., 2000; Gicquel et al., 2009; Insausti et al., 2010; Jin et al., 2015), decrease of scarring (Rowe et al., 1997; Tseng et al., 1999; Koh et al., 2007; Santanna et al., 2016), low immunogenicity (Kubo et al., 2001; Szekeres-Bartho, 2002; Hortensius et al., 2016; Magatti et al., 2018), antimicrobial (Tehrani et al., 2013; Mao et al., 2017, 2018; Yadav et al., 2017; Ramuta et al., 2020b), anti- and pro-angiogenic properties (Hao et al., 2000; Niknejad et al., 2013), recent studies demonstrated that hAM also possesses anticancer properties (Magatti et al., 2012; Mamede et al., 2014, 2015, 2016; Niknejad et al., 2014, 2016; Bu et al., 2017; Riedel et al., 2019; Ramuta et al., 2020a). Moreover, recently we showed that hAM scaffolds hinder the growth and invasive potential of MIBC cells (Ramuta et al., 2020a). However, when considering the application of hAM scaffolds in urology, their handling might be rather difficult. Hence, to facilitate the translation of hAM from bench to bedside, the development of novel hAM-derived preparations (e.g., hAM homogenate) that would allow a more straightforward administration is crucial. Therefore, the aim of our study was to investigate the anticancer activity of hAM homogenates on 2D and 3D *in vitro* models of NMIBC and MIBC.

## Materials and Methods

### Cell Cultures

Human bladder invasive cancer urothelial (T24) cells, papillary cancer urothelial (RT4) cells and low-metastatic breast cancer (MCF7) cells were purchased from ATCC (Manassas, VA, United States), seeded at a seeding density of 5 × 10^4^ cells/cm^2^ and cultured in a 1:1 mixture of A-DMEM medium (Gibco, Thermo Fisher Scientific, Waltham, MA, United States) and F12 (Sigma-Aldrich, St. Louis, MO, United States), supplemented with 5% fetal bovine serum (FBS) (Invitrogen, Carlsbad, CA, United States), 4 mM GlutaMAX (Gibco, Thermo Fisher Scientific, Waltham, MA, United States), 100 μg/ml streptomycin, and 100 U/ml penicillin (Thermo Fisher Scientific, United States). Human mammary gland non-tumorigenic (MCF10a) cells were obtained from ATCC (Manassas, VA, United States), seeded at a seeding density of 5 × 10^4^ cells/cm^2^ and cultured in MEBM^TM^ Basal Medium (CC-3151, Lonza, Basel, Switzerland) supplemented with MEGM^TM^ SingleQuots^TM^ Supplement Pack (CC-4136, Lonza, Basel, Switzerland) and 5% horse serum (HS) (Gibco, Thermo Fisher Scientific, Waltham, MA, United States).

The use of porcine urinary bladders for preparation of primary urothelial cells was approved by the Veterinary Administration of the Slovenian Ministry of Agriculture and Forestry in compliance with the Animal Health Protection Act and the Instructions for Granting Permits for Animal Experimentation for Scientific Purposes. Normal porcine urothelial (NPU) cells were established as previously described (Kreft et al., 2005; Visnjar et al., 2012; Visnjar and Kreft, 2015). The NPU cells were seeded at a seeding density of 1 × 10^5^ cells/cm^2^ and maintained in a 1:1 mixture of MCDB153 medium (Sigma-Aldrich, St. Louis, MO, United States) and Advanced Dulbecco’s modified essential medium (Invitrogen, Carlsbad, CA, United States), supplemented with (final concentrations are shown) 0.1 mM phosphoethanolamine (Sigma-Aldrich, St. Louis, MO, United States), 15 μg/ml adenine (Sigma-Aldrich, St. Louis, MO, United States), 0.5 μg/ml hydrocortisone (Sigma-Aldrich, St. Louis, MO, United States), 5 μg/ml insulin (Sigma-Aldrich, St. Louis, MO, United States), 2 mM GlutaMAX (Gibco, Thermo Fisher Scientific, Waltham, MA, United States), 100 μg/ml streptomycin, 100 U/ml penicillin (Thermo Fisher Scientific, United States) and 2.5% FBS. Upon reaching confluence, NPU cells were cultured in the serum-free medium, supplemented with a calcium concentration (CaCl_2_) of 2.5 mM for additional 3 weeks. All cell cultures were maintained at 37°C in a humidified atmosphere at 5% CO_2_.

All of the cell cultures were maintained in culture media containing antibiotics, while the assays described in sections “Cell detachment assay,” “Cell Attachment Assay,” “Analysis of the Effect of hAM Homogenate on the Proliferation Rate of Cancer Urothelial Cells,” “Western Blot Analysis,” “Scanning and Transmission Electron Microscopy,” and “Analysis of the Effect of hAM Homogenate on the 3D Architecture of Bladder Cancer Urothelial Spheroids” were performed using the antibiotics-free culture media.

### Preparation of Human Amniotic Membrane Homogenate

**FIGURE 1:**
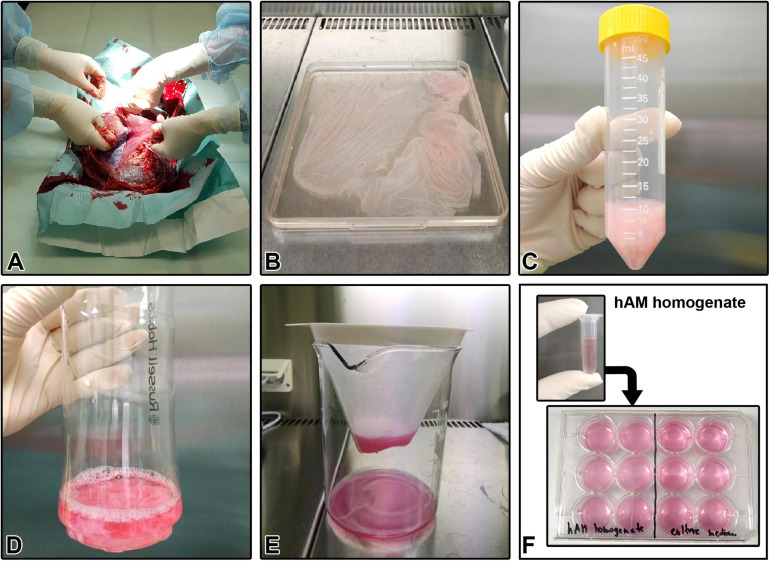
Human amniotic membrane (hAM) homogenate preparation protocol. **(A)** Separation of the hAM from human chorionic membrane (hCM). **(B)** Washing the hAM in sterile PBS. **(C)** Measuring the volume of hAM pieces. **(D)** Addition of an appropriate culture medium to the hAM pieces in the ratio of 1:4. **(E)** Filtration of hAM homogenate through sterile nylon membrane filter with pore size <1 mm, after completed homogenization. **(F)** Cryopreserved hAM homogenate is used for further experiments.

The studies involving human participants were reviewed and approved by the National Medical Ethics Committee of the Republic of Slovenia. The hAM homogenate was prepared as previously described (Šket et al., 2019; Ramuta et al., 2020b). Briefly, 32 hAMs were obtained at the time of elective Cesarean sections from healthy volunteers (age 25–38 years old) who previously signed a written informed consent form. All volunteers were serologically negative for hepatitis B and C, HIV, and syphilis. Right after delivery, hAM was separated from the chorion, washed with sterile phosphate-buffered saline (PBS), and cut into small pieces. The volume of hAM pieces was measured, and the appropriate culture medium was immediately added in the ratio of 1:4 (one part of hAM pieces and three parts of appropriate culture medium without FBS, HS, antibiotics or CaCl_2_). The mixture was homogenized with two different homogenizers: Russell Hobbs 21350-56 (Failsworth, United Kingdom, 300 W, only one speed up to 24000 rpm), and/or Kinematica Polytron^®^ PT 3100 D (Kinematica, Luzern, Switzerland, 1200 W, speed range 500–30 000 rpm) (Table 1). Furthermore, homogenization with Kinematica Polytron^®^ PT 3100 D was performed at three different speeds: 20000, 10000, and 5000 rpm. The prepared homogenates were filtered through sterile nylon membrane filter with pore size <1 mm, stored at −80°C for up to 6 months and underwent only one freeze-thaw cycle. Prior to each experiment, a sufficient volume of FBS (final concentration of 5%; for cancer cell lines), HS (final concentration of 5%; for MCF10a) or CaCl_2_ (final concentration of 2.5 mM; for NPU cells) was added to the respective hAM homogenates. The homogenate prepared with Russell Hobbs was used in all the experiments and we refer to the so-prepared homogenate as hAM homogenate or RH. In contrast, we refer to the hAM homogenate prepared with Kinematica Polytron^®^ PT 3100 D as PT1 (homogenization at 20000 rpm), PT2 (homogenization at 10000 rpm) and PT3 (homogenization at 5000 rpm). Processing of hAM was performed under sterile conditions in a clean room and laminar flow cabinets of biosafety level 2. All experiments using the hAM homogenate were performed without antibiotics. The main steps of the preparation of hAM homogenate are illustrated in Figure 1.

**TABLE 1:** Specifications of homogenizers used for studying anticancer properties of hAM homogenate.

**Homogenizer**	**Manufacturer**	**Power**	**Speed**	**Blade**
Russell Hobbs 21350-56	Russell Hobbs, Failsworth, United Kingdom	300 W	Up to 24000 rpm (non-adjustable)	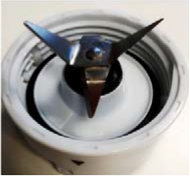
				
Polytron^®^ PT 3100 D	Kinematica, Luzern, Switzerland	1200 W	Adjustable (hAM homogenates were prepared using 20000, 10000, and 5000 rpm)	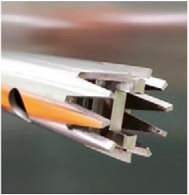
				

### Cell Detachment Assay

To investigate the effect of hAM homogenate on the cell detachment status, the T24, RT4, NPU, MCF7, and MCF10a cells were cultured to confluency and incubated with hAM homogenate for 24-h. Moreover, to further evaluate whether the effect of hAM homogenate on the cell detachment depends on the homogenate preparation (see the protocols in “Preparation of Human Amniotic Membrane Homogenate”) and treatment protocols, T24, RT4 and NPU cells were cultured to confluency and then treated with different homogenate preparations (a) every 24 h for three consecutive days or (b) 2 h per day for three consecutive days. Additionally, we performed experiments with the NPU cells, treated with hAM homogenate every 24-h for a total of seven consecutive days and then cultured in an appropriate culture medium without hAM homogenate for three more weeks. After each treatment, the cells were rinsed three times with culture medium, and 5–10 random bright-field images per well were obtained using an inverted phase-contrast microscope Eclipse E300 (Nikon, Tokyo, Japan). The area of detached cells was analyzed with ImageJ software (Wright Cell Imaging Facility, Toronto, ON, Canada) and presented as a percentage of the total field view area (% of surface area covered). Untreated cells were grown in an appropriate culture medium without the hAM homogenate and served as negative controls. The volume of hAM homogenate or an appropriate culture medium without hAM homogenate was equivalent for all cell types and treatment protocols.

### Cell Attachment Assay

For the attachment assay, T24 and RT4 cells were seeded in a 24-well plate (5 × 10^4^ cells/well) and cultured in the presence or absence (controls) of hAM homogenate for three consecutive days. Every 24 h, fresh hAM homogenate was added to the wells. To quantify the number of attached cells, the hAM homogenate or culture medium was removed. The cells were fixed with 4% formaldehyde (w/v) and stained with Giemsa (Merck, Kenilworth, NJ, United States). The attached cells were imaged by stereo microscope SMZ800N (Nikon, Tokyo, Japan) equipped with MikroCam PRO HDMI 5MP (Bresser Gmbh, Rhede, Germany) and then scanned using the ScanMaker 8700 (Microtek, Hsinchu, Taiwan). The resulting images obtained by the stereomicroscope were converted to an 8-bit binary images using the ImageJ software. The integrated density of the attached cells was quantified and presented as a mean relative intensity (arbitrary units).

### Analysis of the Effect of hAM Homogenate on the Proliferation Rate of Cancer Urothelial Cells

Confluent cultures of T24 and RT4 cells were treated with culture medium (controls) or hAM homogenate for three consecutive days, and the proliferation rate was evaluated after 24, 48, and 72 h of treatment. The proliferation rate was determined using the Click-it Plus EdU Alexa Fluor 488 Imaging Kit (Thermo Fisher Scientific, Waltham, MA, United States) and subsequent analysis was performed with ImageJ software. Briefly, 24 h before the analysis, the T24 and RT4 cells were incubated at 37°C and 5% CO_2_ in the culture medium (controls) supplemented with 10 μM 5-ethynyl-2′-deoxyuridine (EdU) or hAM homogenate supplemented with EdU. Next, the samples were fixed with 4% formaldehyde for 15 min at room temperature (RT), washed three times in 3% bovine serum albumin (BSA) (Thermo Fisher Scientific, Waltham, MA, United States) in PBS and permeabilized by incubation in 0.5% Triton-X-100 (Thermo Fisher Scientific, Waltham, MA, United States) in PBS for 20 min at RT. Afterward, the samples were washed three times in 3% BSA in PBS and incubated for 30 min at RT in the Click-it Plus reaction buffer containing Alexa Fluor picolyl azide according to the manufacturer’s instructions. Next, the samples were washed three times with 3% BSA in PBS and the nuclei were stained using DAPI. The samples were examined using the fluorescence microscope AxioImager.Z1 equipped with ApoTome (Zeiss, Jena, Germany). Furthermore, 5–10 images for each sample were taken and the percentage of proliferating cells was determined with the ImageJ software.

### Western Blot Analysis

Confluent cultures of T24 and RT4 cells were treated with culture medium (controls) or hAM homogenate for 24 h and the expression of cyclin D1 was evaluated. After the treatment, the T24 and RT4 cells were collected and lysed in ice-cold RIPA buffer (Merck, Kenilworth, NJ, United States), containing a cocktail of protease and phosphatase inhibitors (Thermo Fisher Scientific, Waltham, MA, United States). Total protein levels were quantified using the Pierce BCA Protein Assay Kit (Thermo Fisher Scientific, Waltham, MA, United States). Equivalent concentrations of protein (50 mg/lane) were separated using 4–20% Novex WedgeWell Tris-Glycine Gels (Invitrogen, Carlsbad, CA, United States) and then transferred onto a nitrocellulose membrane (Sigma-Aldrich, St. Louis, MO, United States). Then the membranes were blocked in 5% skim milk in 0.1% Tris Buffered saline/Tween 20 (TBS-T) for 2 h at RT and incubated overnight at 4°C with primary antibodies against cyclin D1 (dilution 1:500, sc-753, Santa Cruz Biotechnology, Inc., Dallas, TX, United States) and anti-α-tubulin (dilution 1:2000; T6199, Sigma-Aldrich, St. Louis, MO, United States). The next day, the membranes were washed with TBS-T and immediately incubated for 1 h at RT with secondary antibodies conjugated with horseradish peroxidase (dilution 1:1000, A6154, Sigma-Aldrich, St. Louis, MO, United States). Visualization of the protein bands was performed using the SuperSignal West Pico Chemiluminescent Substrate (Thermo Scientific, Waltham, MA, United States). ImageJ software was used to carry out the densitometric analysis. α-tubulin served as a loading control. Western analyses shown here are representative of three independent experiments.

### Scanning and Transmission Electron Microscopy

Confluent cultures of T24, RT4, and NPU cells were treated with culture medium (controls) or hAM homogenate for 24 and 72 h, respectively. The samples were prepared for scanning and transmission electron microscopy as described previously (Visnjar et al., 2012; Jerman et al., 2014; Ramuta et al., 2020b). Briefly, for scanning electron microscopy the samples were fixed with 2% formaldehyde (w/v) and 2% glutaraldehyde (v/v) in 0.2 M cacodylate buffer (pH 7.4) for 3 h at 4°C. Afterward, the samples were rinsed overnight in the 0.2 M cacodylate buffer and post-fixed in 1% (w/v) osmium tetroxide in 0.2 M cacodylate buffer for 2 h at RT. Next, the samples were dehydrated in a graded series of ethanol, followed by acetone. The samples were then immersed in hexamethyldisilazane (Sigma-Aldrich, St. Louis, MO, United States), air-dried overnight at RT, sputter-coated with gold and examined at 25–30 kV with Vega 3 scanning electron microscope (Tescan, Brno, Czech Republic). For transmission electron microscopy, the samples were fixed with 3% formaldehyde (w/v) and 3% glutaraldehyde (v/v) in 0.1 M cacodylate buffer for 3 h at 4°C. Next, the samples were rinsed overnight in the 0.1 M cacodylate buffer and post-fixed with 2% (w/v) osmium tetroxide for 1 h at RT. Afterward, the samples were incubated in 2% uranyl acetate in H_2_O for 1 h at RT, followed by dehydration in graded series of ethanol and embedding in Epon (Serva Electrophoresis, Heidelberg, Germany). The semithin sections were prepared and stained with toluidine blue and used to localize the position of ultrathin sections and also to count the number of cell layers in *in vitro* models. Then the ultrathin sections were prepared and contrasted with uranyl acetate and lead citrate and examined at the operation voltage 80 kV with the CM100 transmission electron microscope (Philips, Eindhoven, The Netherlands) equipped with the CCD camera (AMT, Danvers, MA, United States).

### Analysis of the Effect of hAM Homogenate on the 3D Architecture of Bladder Cancer Urothelial Spheroids

To prepare the spheroids, the T24 and RT4 cells were seeded in the appropriate culture medium on the ultra-low attachment 96-well plates (Corning, New York, NY, United States) at a seeding density of 100,000 cells per well (T24 cells) or 50,000 cells per well (RT4 cells) and cultured at 37°C and 5% CO_2_ for 96 h. Then the samples were incubated for additional 24 or 72 h in culture medium (controls) or hAM homogenate, fixed in 4% formaldehyde for 60 min at 4°C and then rinsed in PBS for 30 min. Next, to prepare paraffin sections, the samples were dehydrated through a graded series of ethanol into xylene and embedded in paraffin wax and cut with microtome into 7 μm sections. Afterward, the paraffin sections were stained with hematoxylin-eosin and examined with the Eclipse E200 microscope (Nikon, Tokyo, Japan).

### Statistical Analyses

All the experiments were performed with at least 3 independent biological samples of hAM. Within each experiment, three technical replicates were carried out. Statistical analyses were performed by GraphPad Prism 6 (GraphPad Software, La Jolla, CA, United States) or SigmaPlot 12.0 (Systat Software, San Jose, CA, United States) software. The quantified data are presented as mean ± standard error of the mean (SEM). When appropriate, the parametric unpaired two-tailed Student’s *t*-test or the non-parametric Mann–Whitney test was used to compare the statistical difference between two experimental groups. Similarly, one-way analysis of variance (ANOVA) with Tukey’s correction or Kruskal–Wallis with Dunn’s correction for multiple comparisons was used to compare the statistical difference between at least three experimental groups. A *p*-value of <0.05 was considered statistically significant.

## Results

### Effect of hAM Homogenate on Detachment of Various Cancer Cell Lines

**FIGURE 2:**
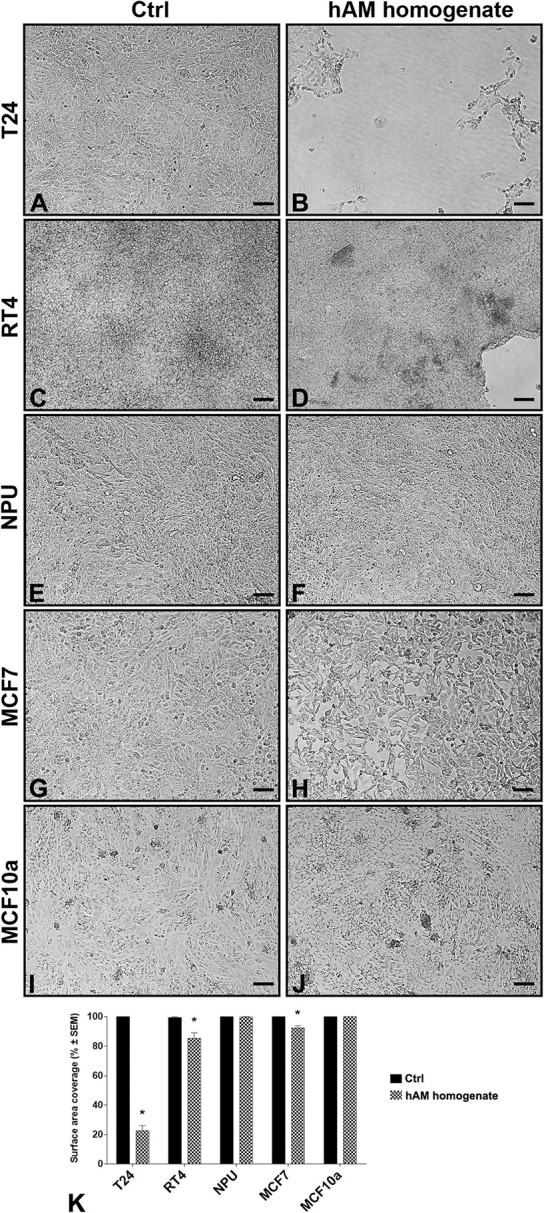
Human amniotic membrane (hAM) homogenate causes detachment of various cancer cell types but not of normal cells. **(A,C,E,G,I)** After 24-h incubation with an appropriate culture medium without hAM homogenate, T24, RT4, NPU, MCF7, and MCF10a cells remained firmly attached to the culture surface. **(B,D,H)** 24-h incubation with hAM homogenate resulted in significant detachment of cancer T24, RT4, and MCF7 cells, **(F,J)** but not of normal NPU and MCF10a cells. The most presentative images are shown. **(K)** The percentage of surface area covered after 24-h treatment with hAM homogenate. The data are presented as mean surface area coverage ± SEM (SEM, the standard error of the mean). Data were obtained from three independent experiments, each performed with a different biological sample of hAM. Within each experiment, three technical replicates were performed. Scale bars: 100 μm. *p < 0.05.

The confluent cultures of T24, RT4 NPU, MCF7, and MCF10a were treated with an appropriate culture medium (controls) and hAM homogenate for 24 h (Figures 2A–J). We found that hAM homogenate caused the detachment of several cancer cell lines. As shown in Figure 2, compared to control cells, the surface area covered with muscle-invasive bladder T24 cells was significantly reduced to 22.7 ± 3.5% (Figures 2B,K). The decrease in surface area coverage was, albeit to a lesser extent, also detectable after 24-h hAM homogenate treatment of non-invasive papillary urothelial RT4 cells (surface area coverage 85.0 ± 3.7%; Figures 2D,K). On the other hand, we did not observe detachment of normal urinary bladder NPU cells (Figures 2F,K). Furthermore, 24-h incubation with hAM homogenate caused detachment of the low-metastatic breast cancer MCF7 cells (surface area coverage 92.5 ± 1.6% (Figures 2H,K), but not of non-tumorigenic breast MCF10a cells, which remained firmly attached to the well surface (Figures 2J,K).

Most of the cancer cells detached from the culture surface, which suggests that hAM homogenate may impair cell-cell and cell-matrix interactions. Taken together, these results demonstrate that cancer urothelial T24 and RT4 cells are more sensitive to hAM homogenate treatment than the breast cancer MCF7 cells. For this reason, biomimetic *in vitro* models of normal and cancerous urothelium were a focus of our further analyses.

### Effect of Different hAM Homogenates and Treatment Protocols on the Detachment of Cancer Urothelial Cells

**FIGURE 3:**
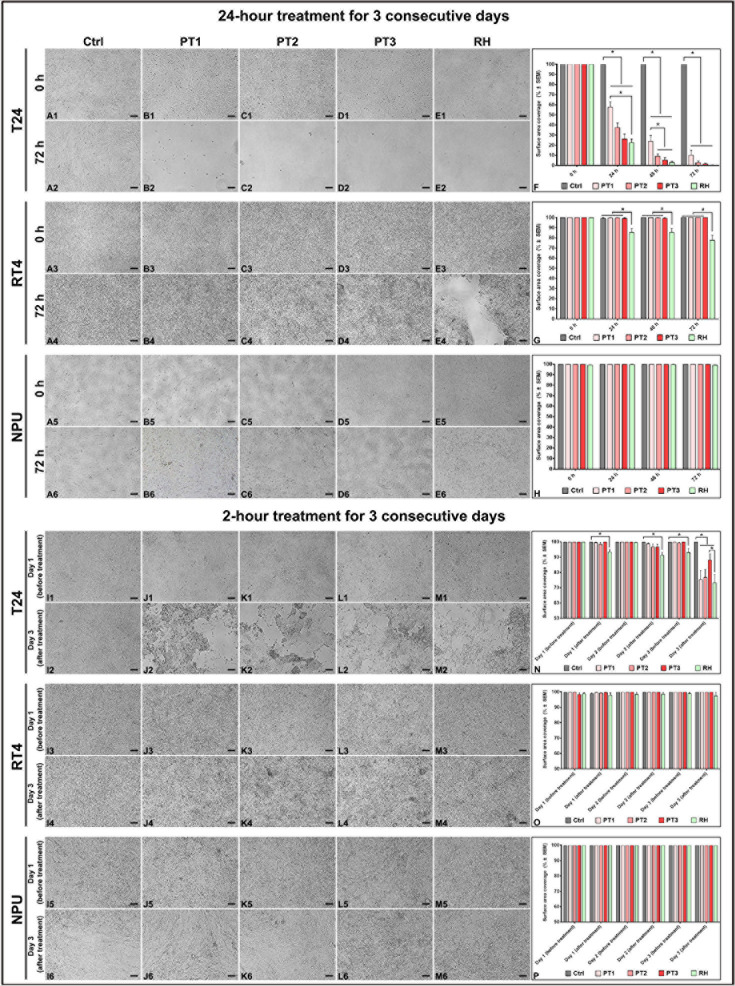
Different hAM homogenate preparations cause detachment of cancer urothelial cells in a time-dependent manner. **(A1–A6)** Confluent cultures of T24, RT4, and NPU cells incubated with an appropriate culture medium without hAM homogenate remained attached to the culture surface after the 72-h treatment period. **(B1–E2)** 72-h treatment with different hAM homogenate preparations resulted in a significant detachment of T24 cells. **(B3–E4)** Only hAM homogenate prepared with Russell Hobbs caused detachment of RT4 cells after the 72-h treatment period. **(B5–E6)** Different hAM homogenate preparations did not cause any detachment of NPU cells after the 72-h treatment period. **(I1–I6)** Confluent cultures of T24, RT4, and NPU cells incubated with an appropriate culture medium without hAM homogenate remained attached to the culture surface on the third day of the 2-h treatment period. **(J1–M2)** Different hAM homogenate preparations caused detachment of T24 cells on the third day of the 2-h treatment period. **(J3–M4)** Confluent cultures of RT4 cells incubated with different hAM homogenate preparations remained attached to the culture surface on the third day of the 2-h treatment period. **(J5–M6)** Different hAM homogenate preparations did not induce detachment of NPU cells in three consecutive days of 2-h treatment. **(F,N)** The percentage of surface area covered with T24 cells after 24- and 2-h treatment for three consecutive days. **(G,O)** The percentage of surface area covered with RT4 cells after 24- and 2-h treatment for three consecutive days. **(H,P)** The percentage of surface area covered with NPU cells after 24- and 2-h treatment for three consecutive days. The quantified data here are presented as mean surface area coverage ± SEM. Data were obtained from three independent experiments, each performed with a different biological sample of hAM. Within each experiment, three technical replicates were performed. Scale bars: 100 μm. *p < 0.05.

To test whether different preparations of hAM homogenate and treatment protocols have an effect on the amount of cell detachment, we treated confluent T24 (Figures 3A1–E1), RT4 (Figures 3A3–E3), and NPU cells (Figures 3A5–E5) with four different types of hAM homogenates (PT1, PT2, PT3 and RH) every 24 h for three consecutive days. Control cells were incubated in culture medium only. Our results showed that following the 24-h treatment with PT1, PT2, PT3, and RH, the surface area covered with T24 was 52.6 ± 5.3%, 37.4 ± 4.8%, 26.2 ± 4.8%, and 22.7 ± 3.5%, respectively (Figure 3F). After 48-h treatment, we observed a similar decreasing trend in the total surface area coverage of T24 cells. The surface area coverage of T24 cells treated with PT1, PT2, PT3, and RH decreased to 21.8 ± 5.1%, 9.0 ± 2.6%, 5.5 ± 2.2%, and 3.1 ± 1.3%, respectively (Figure 3F). Noticeably, we observed the highest effect of hAM homogenate-based cell detachment after 72-h treatment. The surface area coverage of T24 cells treated with PT1 (Figures 3B2,F), PT2 (Figures 3C2,F), PT3 (Figures 3D2,F), and RH (Figures 2E2,F) was 10.0 ± 4.4%, 2.7 ± 1.7%, 1.4 ± 0.8%, and 0.5 ± 0.1%, respectively. Throughout the 72-h treatment period, untreated control T24 cells remained firmly attached to the culture surface (Figures 3A2,3F). What stands out in Figure 3G is that only hAM homogenate prepared with RH had any effect on the surface area coverage of RT4 cells. Our results showed that after 24-, 48-, and 72-h treatment with RH, the surface area covered with RT4 cells, decreased to 85.4 ± 3.7%, 85.5 ± 4.0%, and 77.5 ± 5.1%, respectively (Figures 3E4,G). On the other hand, during the whole 72-h treatment period, RT4 cells treated with PT1, PT2, and PT3 (Figures 3B4–D4,G) remained attached to the culture surface like the control RT4 cells (Figures 3A4,G). In contrast to bladder cancer cells, our study revealed that the normal bladder NPU cells remained firmly attached to the culture surface after 24-, 48-, and 72-h treatment with different hAM homogenate preparations or an appropriate culture medium (Figures 3A6–E6,H). Furthermore, we also showed that NPU cells remained attached following the 7-day treatment of confluent NPU with hAM homogenate prepared with RH or at any time during the following 21 days in the appropriate culture medium. These results additionally confirm that hAM homogenate does not affect detachment, morphology and viability of NPU cells (Supplementary Figure 1).

We next aimed to determine whether 2-h treatment with hAM homogenate would be sufficient to trigger cell detachment. To do so, we treated confluent T24 (Figures 3I1–M1), RT4 (Figures 3I3–M3), and NPU cell cultures (Figures 3I5–M5) with the same hAM homogenate preparations that we previously mentioned, for 24 h/day for three consecutive days. Control cells were incubated in a culture medium only. Our results showed that RH caused detachment of T24 cells after 2 h on the first day of treatment (surface area coverage 93.4 ± 1.7%) (Figure 3N). Furthermore, we observed a similar decreasing trend after the second day of treatment with RH. The total surface area covered with T24 cells decreased to 91.3 ± 2.0% (Figure 3N). The most significant detachment of T24 cells was seen after the third day of treatment (surface area coverage 73.2 ± 5.3%) (Figure 3M2). hAM homogenates prepared with the Polytron homogenizer caused detachment of T24 cells on the third day of the 2-h treatment. Namely, the surface area coverage of T24 cells treated with PT1, PT2, and PT3, decreased to 75.4 ± 5.8%, 76.8 ± 5.2%, and 88.2 ± 4.2%, respectively (Figures 3J2–M2,N). Untreated control T24 cells remained firmly attached to the culture surface throughout the treatment period (Figures 3I2,N). Furthermore, our study revealed that RT4 cells, treated with PT1, PT2, PT3, and RH remained attached to the culture surface in the same fashion as control RT4 cells, as the surface area coverage did not drop below 97.8% at any time during the 2-h treatment period (Figures 3I4–M4,O). Similarly, the percentage of the surface area covered with NPU cells remained unaltered after the consecutive 2-h treatment period with different hAM homogenate preparations or an appropriate culture medium (Figures 3I6–M6,P).

Taken together, we demonstrated that the potency of the hAM homogenate varied between the preparations and the treatment protocols used. We showed that hAM homogenate prepared with Russell Hobbs has the greatest effect on the detachment of urinary bladder cancer T24 and RT4 cells. Therefore, hAM homogenate prepared with Russell Hobbs was used for the remainder of the study.

### Effect of hAM Homogenate on the Attachment Capacity and the Growth Dynamics of Cancer Urothelial Cells

**FIGURE 4:**
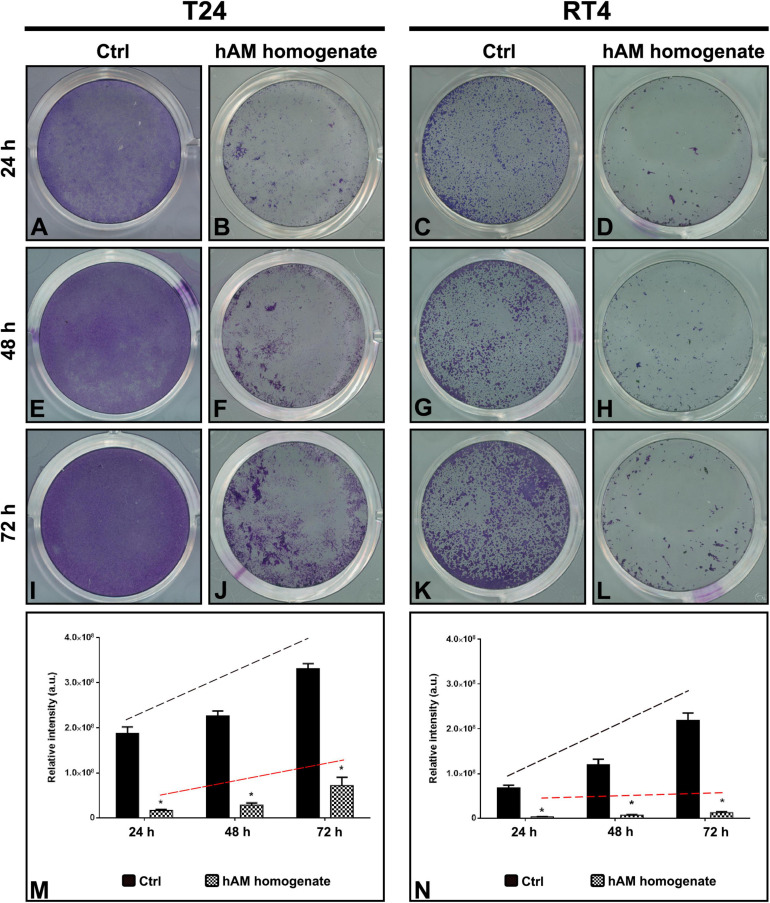
Human amniotic membrane (hAM) homogenate inhibits the cell attachment of T24 and RT4 cells and hinders their growth dynamics. **(A,B)** hAM homogenate significantly reduced the ability of T24 cells to attach to the culture surface after 24-h incubation. **(C,D)** hAM homogenate significantly reduced the ability of RT4 cells to attach to the culture surface after 24-h incubation. **(E,F,I,J)** hAM homogenate strongly inhibited the growth dynamics of the adhered T24 cells 48- and 72-h after the cell seeding. **(G,H,K,L)** hAM homogenate strongly inhibited the growth dynamics of the adhered RT4 cells 48- and 72-h after the cell seeding. **(M,N)** Quantitative analysis of the relative intensity of adherent T24 and RT4 cells. The regression line of the untreated cells (black dashed line) has a steeper upward tilt in comparison with the regression line of cells treated with hAM homogenate (red dashed line). The quantified data here is presented as a mean relative intensity ± standard error of the mean (SEM). Data were obtained from three distinct experiments, each performed with a different biological sample of hAM. Within each experiment, three technical replicates were carried. *p < 0.05.

Next, we aimed to evaluate the effect of hAM homogenate on the cancer urothelial cell attachment capacity. For this reason, we seeded and cultured T24 and RT4 cells in the presence or absence of hAM homogenate for three consecutive days. By measuring the staining intensity of the adherent cells, we showed that hAM homogenate significantly reduced the ability of T24 and RT4 cells to attach to the culture surface after 24-h incubation, in comparison with the untreated control cells (Figures 4A–D,M,N). Furthermore, we also monitored the cell growth for 48 and 72 h after the initial cell seeding (Figures 4E–L,M,N). By comparing the slopes of the regression lines, we observed that once the bladder cancer cells attached to the culture surface, hAM homogenate hindered their growth dynamics and inhibited their spreading potential. Moreover, this inhibitory effect was present in both bladder cancer models, but even more pronounced in the case of papillary urothelial neoplasm RT4 cells.

### Effect of hAM Homogenate on the Proliferation Rate of Cancer Urothelial Cells and Expression of Cyclin D1

**FIGURE 5:**
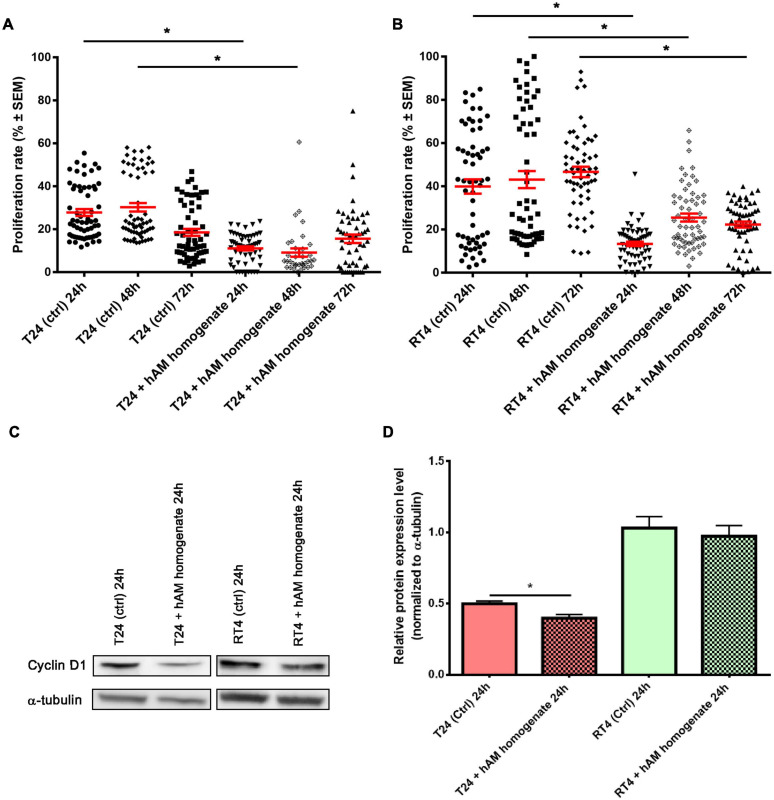
Human amniotic membrane (hAM) homogenate decreases proliferation of T24 and RT4 cells and downregulates the expression of cyclin D1 in T24 cells. **(A)** The proliferation of T24 cells was decreased after 24, 48, and 72 h of treatment with hAM homogenate. **(B)** The proliferation of RT4 cells was decreased after 24, 48, and 72 h of treatment with hAM homogenate. **(C,D)** Western blot analysis of cyclin D1 and α-tubulin expression in T24 and RT4 cells treated with culture medium (ctrl) or hAM homogenate. The western blot analysis showed significant decrease in the expression levels of cyclin D1 after 24-h treatment with hAM homogenate in T24 cells. In RT4 cells, on the other hand, hAM homogenate induced slight but not significant decrease of cyclin D1 expression. All data shown here were obtained from at least three independent replications of experiments using three biological samples of hAM; each experiment was performed in two technical repeats for each condition. Bars represent mean ± SEM. *p < 0.05.

Taking into account the results presented in the previous section, we further investigated the impact of hAM homogenate treatment for three consecutive days on the proliferation rate of T24 and RT4 cells. hAM homogenate decreased proliferation of T24 cells for 16.7% on day 1 (*p* < 0.05), for 21.1% on day 2 (*p* < 0.05), and for 3.0% on day 3 (*p* = 0.1535) of treatment (Supplementary Table 1 and Figure 5A). Similarly, the hAM homogenate also decreased the proliferation of RT4 cells, namely, for 26.6% on day 1 (*p* < 0.05), for 17.6% on day 2 (*p* < 0.05), and for 24.3% on day 3 (*p* < 0.05) (Supplementary Table 1 and Figure 5B). Furthermore, we performed a western blot analysis, which showed a decrease in the expression level of cyclin D1 in T24 cells after 24-h treatment with hAM homogenate (Figures 5C,D). The western blot analysis of RT4 cells showed a slight decrease in the expression levels of cyclin D1 after 24-h treatment with hAM homogenate, but the difference was not statistically significant (Figures 5C,D).

### hAM Homogenate Adheres to the Surface of Cancer Urothelial Cells and Not of Normal Urothelial Cells

**FIGURE 6:**
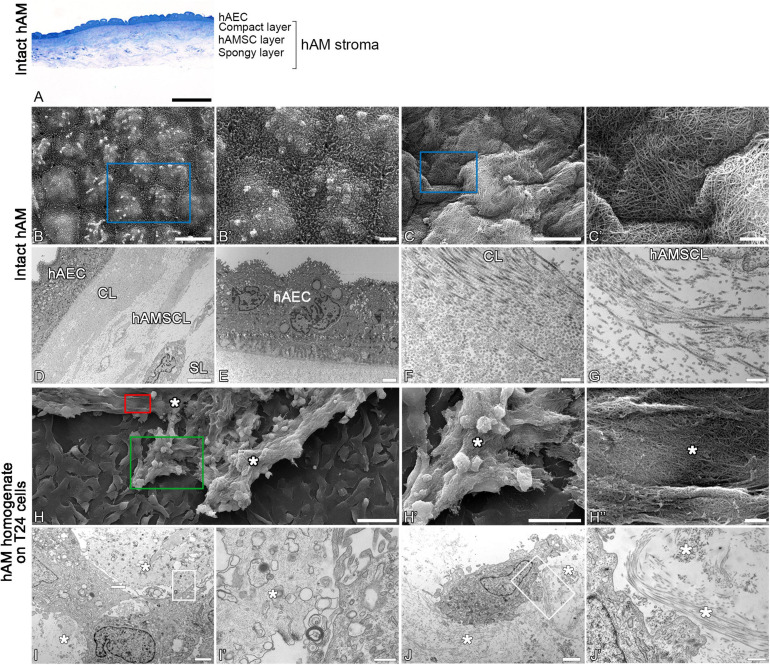
Structure of hAM and hAM homogenate. **(A)** Intact hAM is comprised of hAEC and hAM stroma, which is further divided into the compact layer, hAMSC layer and spongy layer. **(B,B’,D,E)** hAEC are of cuboidal form, are well connected and exhibit numerous microvilli at the apical surface. **(C,C’,F,G)** The fibers that form the extracellular matrix of hAM’s stroma are tightly interwoven. **(D)** The hAM stroma is further divided into the compact layer (CL), hAMSC layer (hAMSCL), and spongy layer (SL). **(H–J)** The hAM homogenate applied to T24 cells. The hAM homogenate (white asterisks) consisted of the tightly interwoven fibers of the extracellular matrix of hAM’s stroma and hAM-derived cells. The hAM homogenate applied to T24 cells adhered to the cell surface. The blue rectangles in panels **(B,C)** mark the areas enlarged in panels **(B’,C’)**. The green rectangle marks the area enlarged in panel **(H’)**; the red rectangle marks the area enlarged in panel **(H”)**. White framed areas in panels **(I,J)** mark the areas enlarged in panels **(I’,J’)**. **(A)** 100 μm, **(B,C)** 10 μm, **(H)** 50 μm, **(H’)** 25 μm, **(D)** 6 μm, **(B’,C,E,H”,I,J)** 2 μm, **(J’)** 600 nm, **(F,G,I’)** 400 nm.

Using the scanning and transmission electron microscopy, we first examined the ultrastructure of intact hAM, which is comprised of a monolayer of hAEC, basal lamina and hAM stroma (Figures 6A–G). Then we investigated the ultrastructure of hAM homogenate, which is a mixture of hAM cells, mainly hAEC and hAMSC, and hAM’s extracellular matrix (ECM). As the hAM homogenate represents the majority of the hAM (Figures 6A,C,C’,F,G), we detected large amounts of hAM’s ECM also in hAM homogenates (Figures 6H–J’).

**FIGURE 7:**
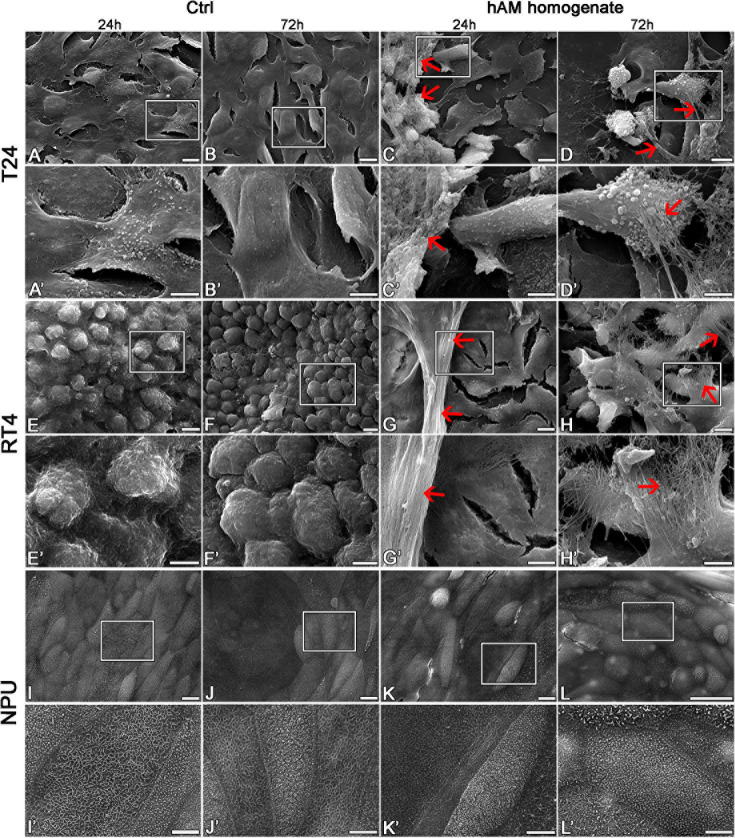
The hAM homogenate adheres to the surface of T24 and RT4 cells. **(A–B**’) The T24 cells incubated in culture medium for 24 or 72 h had mesenchymal morphology and there were large intercellular spaces between the cells. **(C–D**’) The 24- and 72-h incubation in hAM homogenate did not significantly affect the morphology of T24 cells. The hAM homogenate adhered to the surface of T24 cells and a large portion of cells were covered with it. **(E–F**’) The RT4 cells incubated in culture medium for 24 or 72 h had epithelial morphology and were well connected. **(G–H**’) The 24- and 72-h incubation in hAM homogenate did not significantly affect the morphology of RT4 cells. The hAM homogenate adhered to the surface of RT4 cells and a large portion of cells were covered with it. **(I–J**’) The NPU cells incubated in culture medium for 24 or 72 h retained the apical topography of well-differentiated normal urothelial cells. The apical plasma membrane appeared as ropy and rounded ridges, and rarely microridges. **(K–L**’) The 24- and 72-h incubation in hAM homogenate did not significantly affect the morphology of NPU cells and the hAM homogenate did not adhere to the surface of NPU cells. All data shown here were obtained from at least three independent replications of experiments using three biological samples of hAM; each experiment was performed in 1–2 technical repeats for each condition. Red arrows–hAM homogenate. Frames in panels **(A–L)** mark enlarged areas shown in panels **(A**’**–L**’). Scale bars: **(A–L)** 10 μm, **(A**’**–L**’) 5 μm.

**FIGURE 8:**
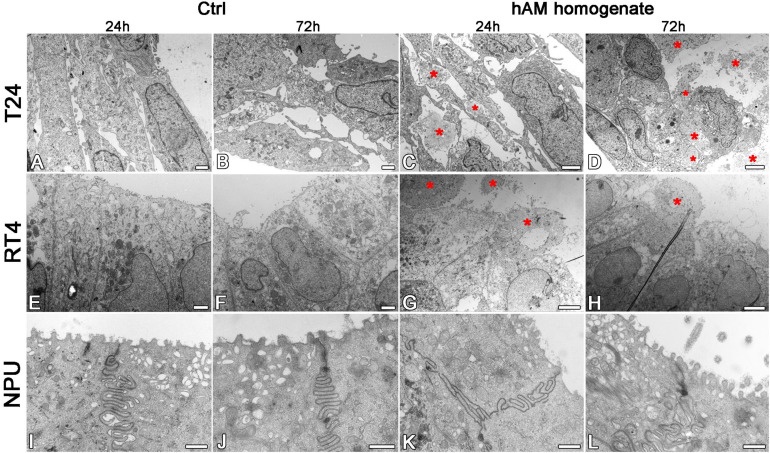
The hAM homogenate adheres to the surface of T24 and RT4 cells, but not the NPU cells, and incorporates between T24 cells. **(A,B)** The T24 cells incubated in culture medium for 24 or 72 h had mesenchymal morphology and there were large intercellular spaces between the cells. **(C,D)** The hAM homogenate (red asterisks) adhered to the surface of T24 cells and incorporated into the intercellular spaces. **(E,F)** The RT4 cells incubated in culture medium for 24 or 72 h had epithelial morphology and were well connected. **(G,H)** Incubation in hAM homogenate for 24 or 72 h had no significant effect on RT4 cell morphology. The hAM homogenate (red asterisks) adhered to the surface of RT4 cells. Some RT4 cells begin to desquamate. **(I,J)** The NPU cultures incubated in culture medium 24 or 72 h retained the typical ultrastructure of well-differentiated normal urothelial cells. **(K,L)** Incubation in hAM homogenate for 24 or 72 h had no significant effect on NPU cell morphology, and the hAM homogenate did not adhere to the surface of NPU cells. All data shown here were obtained from at least three independent replications of experiments using three biological samples of hAM; each experiment was performed in 1–2 technical repeats for each condition. Scale bars: **(A,B)** 1 μm, **(C)** 2 μm, **(D)** 4 μm, **(E,F)** 10 μm, **(G)** 8 μm, **(H)** 6 μm, **(I,L)** 600 nm.

Then we investigated the effect of hAM homogenate on the morphology, apical surface and ultrastructure of T24, RT4, and NPU cells. Our results showed that T24 cells incubated in a culture medium, have a mesenchymal morphology characteristic of cancer cells, and there are large intercellular spaces between the multiple layers of poorly connected cells (Figures 7A–B’, 8A,B). The T24 cells incubated in hAM homogenate retained the mesenchymal morphology and we observed that the hAM homogenate covered a significant portion of the cells (Figures 7C–D’). As the T24 cells were thoroughly rinsed with culture medium prior to the fixation, this suggests that the remaining hAM homogenate adhered strongly to the surface of T24 cells (Figures 7C–D’). Furthermore, hAM homogenate was found not only adhered to the apical surface of the superficial layer of T24 cells, but also incorporated into the large intercellular spaces of T24 cultures (Figures 8C,D).

The RT4 cells incubated in the culture medium were well connected, without larger intercellular spaces, arranged in multiple layers and exhibited epithelial morphology (Figures 7E–F’, 8E,F). On the other hand, the RT4 cells incubated in hAM homogenate retained their epithelial morphology, but were covered with a significant amount of hAM homogenate (Figures 7G–H’) that was strongly adhered to their surface, despite the multiple rinses, which were performed prior to the fixation of the cells. However, unlike in the T24 cells, hAM homogenate was adhered only to the superficial cell layers of RT4 cells and was not incorporated between lower cell layers. For this reason, we then sought to investigate whether hAM homogenate affected the total number of cell layers. We quantified the maximum and the minimum number of cell layers using the semithin sections of treated and untreated RT4 samples after the 72-h treatment period (Supplementary Figure 2). Our results showed that the number of cell layers of RT4 cells decreased after the 72-h treatment with hAM homogenate (*p* = 0.071). Results of the light microscopy (analysis of semithin sections) and transmission electron microscopy indicate that treatment of RT4 cells with hAM homogenate leads to desquamation of the RT4 cells.

The apical plasma membrane of NPU was formed mainly into ropy and rounded ridges, some of the NPU cells formed also microridges, the ultrastructural characteristic of terminally differentiated urothelial cells (Figures 7I–J’, 8I,J). The hAM homogenate did not affect the morphology of the NPU cells and in contrast to the effect on cancer cells, the hAM homogenate did not adhere to the surface of NPU cells (Figures 7K–L’, 8K,L).

### Effect of hAM Homogenate on the Architecture of T24 and RT4 Spheroids

**FIGURE 9:**
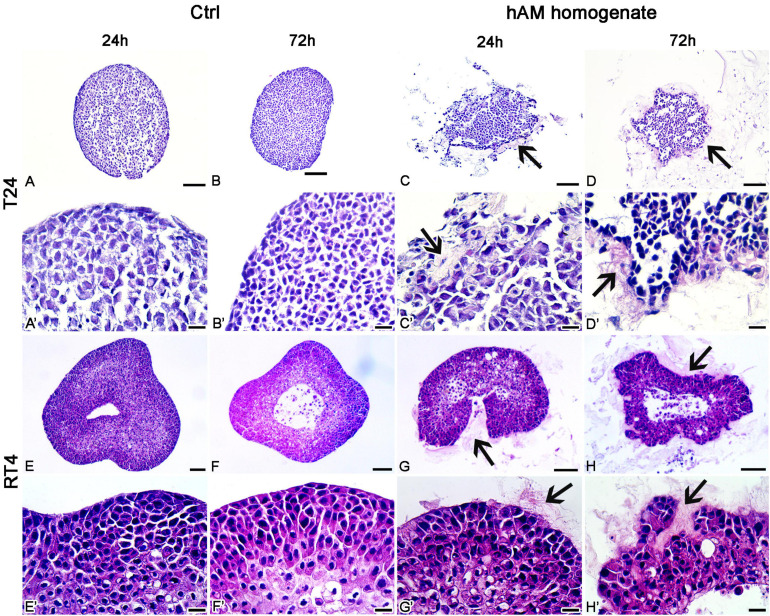
Human amniotic membrane (hAM) homogenate disrupts the architecture of T24 and RT4 spheroids. **(A–B**’) The T24 spheroids incubated in culture medium retained a compact spherical structure. **(C–D**’) 24- and 72-h incubations in hAM homogenate resulted in the disrupted 3D structure of T24 spheroids. hAM homogenate adhered to the surface of T24 spheroids and was in some parts even incorporated into the spheroid. **(E–F**’) The RT4 spheroids incubated in culture medium retained a compact spherical structure. **(G–H**’) 24- and 72-h incubations in hAM homogenate resulted in the disrupted 3D structure of RT4 spheroids as the hAM homogenate adhered to the surface of RT4 spheroids and was in some parts incorporated into the spheroid. All data shown here were obtained from at least three independent replications of experiments using three biological samples of hAM; each experiment was performed in at least three technical repeats for each condition. Arrows–hAM homogenate. Scale bars: **(A–D, E–H)** 100 μm, **(A**’**–D**’**, E**’**–H**’) 20 μm.

The T24 and RT4 spheroids, incubated in a culture medium, retained a compact spherical structure (Figures 9A–B’,E–F’). On the other hand, the incubation of T24 and RT4 spheroids in hAM homogenate resulted in a disrupted 3D structure already after the 24 h of incubation and the effect was even more pronounced after 72 h of incubation. Namely, the treatment with hAM homogenate led to larger intercellular spaces between cancer cells, which occurred to a greater extent in the spheroids of T24 cells than RT4 cells. Furthermore, the hAM homogenate adhered to the surface of T24 and RT4 and was also incorporated into the spheroids of T24 and RT4 cells, which resulted in an even more disrupted 3D structure (Figures 9C–D’,G–H’).

## Discussion

### Different hAM Homogenate Preparations Cause Detachment of Cancer Urothelial Cells in a Time-Dependent Manner

In the present study, we showed that hAM homogenate induced gradual detachment of cancerous cells from the surface, while it did not affect the normal cells. Our results showed that the degree of detachment of cancer urothelial T24 and RT4 cells was greater than that of breast cancer MCF7 cells. On the other hand, NPU cells remained attached to the culture surface and without any observable changes in their morphology even after a 7-day treatment with hAM homogenate. Taken together, we conclude that the detachment of cancer cells was hAM homogenate-specific. Furthermore, we observed that most of cancer cell detachment came after the washing step, which suggests that hAM homogenate may impair the cell-cell and/or cell-matrix interactions. However, further studies are required to determine the exact mechanism of action that leads to cancer cell detachment.

Furthermore, we showed that the extent of cancerous urothelial cell detachment differs between the treatment protocols with hAM homogenate. We observed the highest effect of hAM homogenate-based cell detachment after 24-h treatment for three consecutive days. Moreover, we demonstrated that 2-h treatment for three consecutive days is sufficient to trigger detachment albeit to a lesser extent. In addition to the treatment protocols, the potency of hAM homogenate on the cancer urothelial cell detachment varied between the used preparations. We observed that treatment with hAM homogenate prepared with Russell Hobbs resulted in the highest number of detached cancer cells. We hypothesize that the differences in the effect might be attributed to the method of homogenization. Namely, the Russell Hobbs homogenizer uses the rotary blades to homogenize the tissue, whereas Polytron uses dispersing aggregate that works based on the rotor-stator principle to homogenize the tissue. The rotor-stator homogenizers disrupt the tissue using the hydraulic and mechanical shear and cavitation (Dhankhar, 2014). However, they have a tendency to cause foaming, which might result in low yield and denaturation of anticancer molecules that are released from the homogenized tissue (Vitzthum, 2010; Dhankhar, 2014). Another factor that might influence the preservation of the anticancer molecules is the motor input power of the homogenizers. Russell Hobbs homogenizer has a 300-Watt motor, whereas Polytron^®^ PT 3100 D has a powerful 1200-Watt motor. On the basis of our results, we hypothesize that homogenization of hAM with low power rotary-blades preserves the anticancer activity of hAM homogenate. Further studies should determine the impact of homogenizing on the anticancer molecules’ preservation and maintenance of their function.

### hAM Homogenate Decreases the Adhesion of Cancer Urothelial Cells and Impedes Their Growth Dynamics

Cancer cell adhesion is a process, which plays a major role during the metastasis of primary tumor cells (Bendas and Borsig, 2012). We showed that when incubated with hAM homogenate, the ability of a suspension of T24 cells and RT4 cells to attach to the culture surface was compromised. We postulate that hAM homogenate may coat the surface of the cells and consequently prevent the binding of the cancer cell to the substratum. Furthermore, it is also possible that when seeded with hAM homogenate, the majority of T24 and RT4 cells bind to the components of the hAM ECM and hence are not able to attach to culture surface. We, therefore, assume that reduced cancer cell adhesion to culture surface after treatment with hAM homogenate stem from the fact that hAM has already been proven to be an excellent scaffold (Niknejad et al., 2008; Jerman et al., 2014). In addition, our research group has already shown that when seeded on hAM scaffolds, muscle-invasive bladder T24 cells lose their invasive potential, which further supports the argument that hAM may impede cancer progression and metastasis (Ramuta et al., 2020a).

Even though hAM homogenate significantly inhibited the adhesion, a small number of bladder cancer cells were still able to attach to the culture surface. Nevertheless, we demonstrated that hAM homogenate significantly slowed down the proliferation rate of the cells that did attach. This inhibitory effect was even more noticeable on the RT4 cells, which underlines the potential of hAM homogenate for battling the highly recurrent papillary NMIBC and flat (carcinoma *in situ*–CIS) bladder tumors.

### hAM Homogenate Decreases the Proliferation of Cancer Urothelial Cells

One of the key characteristics of cancer cells is their ability to proliferate uncontrollably. Our results show that hAM homogenate severely diminishes the proliferation rate of T24 and RT4 cells. This result is also in line with our previous study, in which we demonstrated that hAM scaffolds diminish the proliferation of T24 cells (Ramuta et al., 2020a). Next, we investigated the effect of hAM homogenate on the expression of cyclin D1, which is essential for G_1_/S phase transition and its overexpression had been recorded in a large proportion of human cancers, including bladder cancer (Tut et al., 2001; Musgrove et al., 2011; Choi et al., 2012; Kopparapu et al., 2013). Interestingly, we demonstrated that even though the basal level of cyclin D1 was significantly higher in the RT4 cells, hAM homogenate significantly downregulated the expression of cyclin D1 only in the T24 cells. However, in RT4 cells, hAM homogenate induced slight but not significant decrease of cyclin D1 expression (Figure 5). Hence, we hypothesize that hAM homogenate affects various cell cycle proteins, and that effect depends on the type of cancer cell, which is supported by several studies showing that hAM affects a plethora of cell cycle-related proteins in cancer cells. Namely, it has been shown on human cancer cell lines of hematopoietic origin (KG1, KG1a, Jurkat and U937 cell lines) and non-hematopoietic origin (Girardi, HeLa, and Saos cell lines) (Magatti et al., 2012) and on human ovarian cancer cell line SK-OV-3 (Bu et al., 2017) that hAM-derived cells induce cell cycle arrest in cancer cells in the G_0_/G_1_ phase (Magatti et al., 2012; Bu et al., 2017). Magatti et al. (2012) demonstrated that hAM-derived cells downregulate the expression of cyclins D2, E1, and H and cyclin-dependent kinases (CDK4, CDK6, and CDK2) and upregulate the negative regulators of cell cycle, such as p15 and p21 (Magatti et al., 2012). Furthermore, Riedel et al. (2019) showed that the conditioned medium of hAM induces cell cycle arrest in the HepG2 human liver cancer cell line in the G_2_/M phase (Riedel et al., 2019), which further supports our hypothesis that hAM homogenate affects multiple targets in the cell cycle of cancer cells. To conclude, we show for the first time that the hAM homogenate decreases proliferation and is also capable of altering the expression of cyclin D1 in cancer urothelial cells. Importantly, we demonstrate that the hAM homogenate affects the expression of cyclin D1 in cancer urothelial cells, as the expression is significantly diminished in the invasive cancer urothelial (T24) cells. On the other hand, the expression of cyclin D1 in the papillary cancer urothelial (RT4) cells treated with hAM homogenate is lower than in untreated cell although, the difference is not statistically significant. Overall, that indicates that different cell signaling pathways might be affected by the hAM homogenate in various cells and further research is needed to elucidate the complex and multimodal activity of hAM homogenate in cancer cells of diverse origins.

### The Extracellular Matrix of hAM Homogenate Firmly Adheres to Bladder Cancer Cells and Disrupts the 3D Structure of Bladder Cancer Spheroids

We have demonstrated that hAM homogenate firmly adheres to bladder cancer cells but not normal urothelium. We attribute this to differences in chemical structure of plasma membranes and membrane fluidity between cancer and normal cells as we have shown previously (Yu et al., 2016; Zalba and Ten Hagen, 2017). Moreover, our research group demonstrated that the amounts of cholesterol and sphingomyelin/cholesterol membrane domains are highly increased in urothelial cancer cells in comparison to normal urothelial cells (Resnik et al., 2015). There are also many variations in the presence of various receptors, for example integrins, which mediate contacts between the ECM and stromal cells or tumor cells and are overexpressed in bladder cancer cells (Grossman et al., 2000; Vallo et al., 2017; Arun et al., 2018; Liu et al., 2020). Therefore, further studies are required to elucidate the mechanism of adherence of hAM homogenate to cancer cells.

Improved targeting is one of the goals when developing novel therapeutic approaches since many of the current chemotherapeutics target all rapidly proliferating cells, which leads to a high level of toxicity of the treatment and severe side effects (Feitelson et al., 2015). Therefore, a great advantage of hAM homogenate is also its ability to adhere to bladder cancer cells but not normal urothelial cells.

To test whether the hAM homogenate has such a profound effect on bladder cancer cells also in the 3D *in vitro* models, we prepared the T24 and RT4 spheroids. Our results show that already after 24 h of incubation in hAM homogenate, the architecture of the bladder cancer spheroids was distorted, and the effect was even more pronounced after 72 h of incubation. Namely, the hAM homogenate was not only adhered to the T24 and RT4 cells on the surface of the spheroid but also penetrated the spheroids. Therefore, the important novelty of our study is that we demonstrate for the first time that hAM homogenate can disrupt the 3D structure of urothelial tumor spheroids. Future studies must assess whether in the *in vivo* conditions the hAM homogenate might decrease hypoxia and angiogenesis in tumors and could also contribute to a more efficient drug delivery in the case of the intravesical application of chemotherapeutics.

### hAM Homogenate and Future Perspectives of Bladder Cancer Treatment

Human amniotic membrane homogenate displays many characteristics that would be beneficial in the treatment of bladder cancer. Its anti-proliferative, anti-adhesive and cell detachment-inducing properties suggest that hAM homogenate could be used as adjuvant intravesical therapy after transurethral resection of NMIBC tumors similarly as intravesical chemotherapy and immunotherapy with BCG are used now. According to the European Organization for Research and Treatment of Cancer (EORTC), patients with high risk NMIBC, which have high probability of progression to MIBC (Sylvester et al., 2006), would benefit from additional intravesical agent besides BCG, as BCG therapy either fails or is contraindicated in certain patients. Immediate intravesical instillation of hAM after transurethral resection of bladder tumor (TURBT) could prevent reimplantation of cancer cells that are present in urine due to surgical procedure, while its anti-proliferative and detachment-inducing actions could target cancer cells in bordering regions between tumor and normal mucosa as well as invisible small satellite tumor nests growing in other parts of bladder mucosa.

Furthermore, we believe future studies should be aimed at investigating the effect of hAM homogenate in combination with cytotoxic anticancer drugs, e.g., cisplatin-based chemotherapeutics that remain the gold standard for the treatment of bladder cancer (Dumont et al., 2019). The hAM homogenate could serve as a drug delivery tool for chemotherapeutics since several studies demonstrated the ability of hAM to uptake antibiotics, nanoparticles and other drugs (Kim et al., 2001; Mencucci et al., 2006; Resch et al., 2010, 2011; Li et al., 2012; Hu et al., 2015; Yelchuri et al., 2017; Francisco et al., 2020; Ramuta et al., 2020b). Moreover, their results show that the uptake of the drug was dose-dependent and occurred rapidly, but the release of the drug from hAM was sustained and lasted for up to several days (Mencucci et al., 2006; Resch et al., 2011; Yelchuri et al., 2017; Sara et al., 2019).

Human amniotic membrane homogenate could also contribute to the regeneration of normal urothelium since it was demonstrated that hAM and hAM-derived preparations promote epithelization and decrease scarring (Koizumi et al., 2000; Insausti et al., 2010; Jin et al., 2015; Rahman et al., 2019; Rana et al., 2020). Moreover, our research group showed that hAM scaffolds enable the development of tissue-engineered urothelium with molecular and ultrastructural properties comparable to that of the native urothelium (Jerman et al., 2014) and that hAM scaffolds enriched with urinary bladder fibroblasts promote the re-epithelization of urothelial injury (Jerman et al., 2020). Additionally, recently we showed that hAM homogenate possesses antimicrobial activity against most common uropathogenic bacteria and multidrug-resistant bacteria associated with urinary tract infections (Šket et al., 2019; Ramuta et al., 2020b, 2021) and as such could prevent urinary tract infections, which are one of the most common bacterial infections in humans and also one of the most common healthcare-associated infections (Flores-Mireles et al., 2015; Wang et al., 2019). Therefore, the use of hAM homogenate could prevent additional complications that could arise during the treatment of bladder cancer.

### Anticancer Activity of hAM-Derived Preparations: Which Derivative Encompasses the Most of the Beneficial Properties of hAM?

Several research groups investigated the anticancer activity of hAM using hAM-derived cells (Jiao et al., 2012; Kang et al., 2012; Magatti et al., 2012; Bu et al., 2017), conditioned medium prepared using hAM-derived cells (Niknejad et al., 2014; Kim et al., 2015) or intact hAM (Niknejad et al., 2014; Modaresifar et al., 2017; Riedel et al., 2019) and hAM extracts (Mamede et al., 2014, 2015, 2016). As already mentioned, hAM-derived cells decrease proliferation of cancer cells of hematopoietic and non-hematopoietic origin and ovarian cancer cell line (Magatti et al., 2012; Bu et al., 2017). Moreover, hAMSC induce apoptosis of C6 glioma cells in *in vivo* BALB/c-nu mice model (Jiao et al., 2012). hAM-derived conditioned medium has been shown to inhibit DNA synthesis, decrease viability and number of hepatocarcinoma cells and interestingly, induce cell cycle arrest in G2/M phase (Riedel et al., 2019). Moreover, Niknejad et al. (2014) demonstrated that hAEC-derived conditioned medium decreases viability of cervical cancer and breast cancer cell lines and induces apoptosis (Niknejad et al., 2014). hAM extracts induce cell morphology alterations, modify oxidative stress environment and cell cycle in hepatocarcinoma cells, affect the metabolism of various cancer cell lines and in some hepatocarcinoma cell lines also lead to cell death (Mamede et al., 2014, 2015, 2016). On the other hand, Kim et al. (2015) showed that hAMSC-derived conditioned medium increased proliferation and migration of breast cancer cells, MCF-7 and MDA-MB-231 (Kim et al., 2015). Our previous study showed that the hAEC and hAMSC in co-culture with T24 cancer cells diminish the proliferation of cancer cells, while the hAM-derived scaffolds altered the growth dynamic of T24 cells, reduced their proliferation, decreased expression of epithelial-mesenchymal transition markers N-cadherin, Snail and Slug. Moreover, despite their muscle-invasive potential, the T24 cells did not disrupt the basal lamina of hAM scaffolds even after 3 weeks in culture (Ramuta et al., 2020a).

These studies show that hAM and hAM-derived preparations are in various ways detrimental to cancer cells. Interestingly, some of the studies show conflicting effects of hAM-derived preparations in cancer cells of different origin, indicating that the mechanism of action might be hAM-derived preparation-specific and cell type-specific. Moreover, studies performed by other research groups included the use of hAM-derived cells and their conditioned medium or extract, but we have shown that the hAM’s ECM is a monumental part of hAM and plays an important role in its anticancer activity (Ramuta et al., 2020a). However, when considering a potential clinical application, the hAM scaffolds might be difficult to handle, especially when considering the application in treatment of urological or breast cancers. Hence, the need for another hAM-derived preparation that would combine hAM-derived cells together with ECM arose.

In this study, we therefore prepared hAM homogenate for investigating the anticancer properties of hAM and for the first time we documented its effect on the morphology, attachment, proliferation, cell cycle and ultrastructure of various cancer cells. We obtained promising results that reveal the capability of hAM homogenate to target several hallmarks of cancer cells. However, it is of the utmost importance that future studies identify the molecules in the hAM homogenate that induce the detrimental effects in cancer cells and then determine their mechanism of action.

## Conclusion

This study demonstrates the multi-targeted anticancer activity of hAM homogenate on several cancer cell lines and reveals its potential to be used in bladder cancer treatment. We hypothesize that if combined with cytotoxic anticancer drugs and applied intravesically, the hAM homogenate could contribute to treatment by (1) promoting detachment of bladder cancer cells and preventing their re-attachment to the urothelium, (2) decreasing proliferation of bladder cancer cells, (3) improving targeting of bladder cancer cells without having a toxic effect on normal urothelial cells and (4) improving drug delivery of cytotoxic agents by disrupting the structure of bladder tumors.

## Data Availability Statement

The raw data supporting the conclusions of this article will be made available on request to corresponding author, without undue reservation.

## Ethics Statement

The studies involving human participants were reviewed and approved by the National Medical Ethics Committee of the Republic of Slovenia and all participants provided their written informed consent to participate in this study. The use of animal tissues was reviewed and approved by Veterinary Administration of the Slovenian Ministry of Agriculture and Forestry in compliance with the Animal Health Protection Act and the Instructions for Granting Permits for Animal Experimentation for Scientific Purposes.

## Author Contributions

AJ, TŽR, and MEK designed the study. AJ, TŽR, LT, ŽS, and MEK performed the experiments. AJ, TŽR, LT, and MEK analyzed and interpreted the results. AJ and TŽR wrote the first draft of the manuscript. All authors were involved in critically revising the manuscript for important intellectual content, had full access to the data, and approved the final manuscript.

## Conflict of Interest

The authors declare that the research was conducted in the absence of any commercial or financial relationships that could be construed as a potential conflict of interest.
