# Supplementary material for: Detrimental Effect of Various Preparations of the Human Amniotic Membrane Homogenate on the 2D and 3D Bladder Cancer In vitro Models
Source: Front Bioeng Biotechnol. 2021 Jun 25;9:690358. doi: 10.3389/fbioe.2021.690358 (PMC8267883; doi:10.3389/fbioe.2021.690358)
Supplement: Supplementary file 1 [file Data_Sheet_1.docx]

***Supplementary material***

**
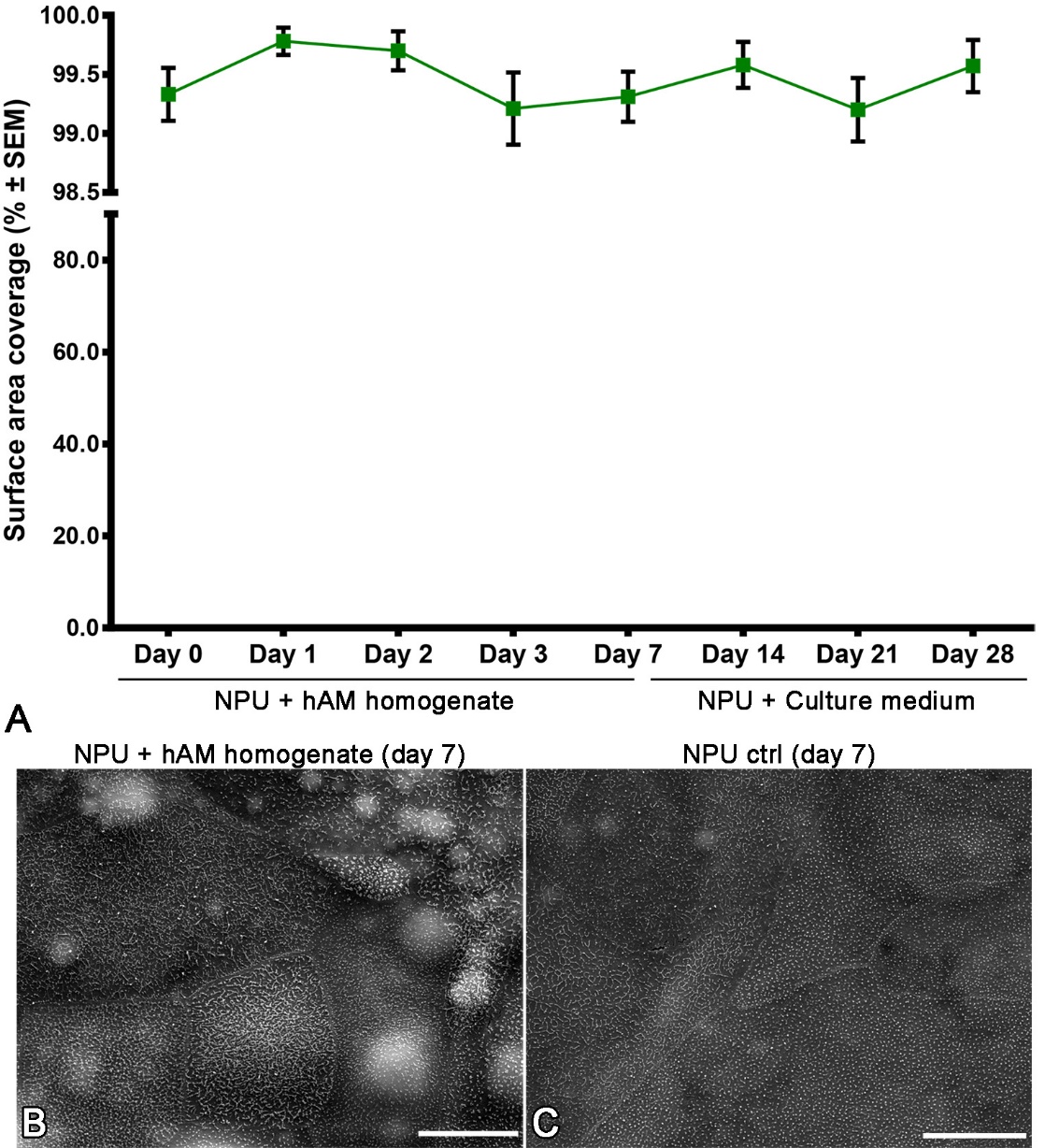
**

**Supplementary Figure S1.** Treatment with hAM homogenate is not detrimental to NPU cells. (A) The 7-day treatment of a confluent NPU cell culture with hAM homogenate did not induce detachment of NPU cells during the treatment or in the following 21 days. (B, C) The 7-day treatment with hAM homogenate did not affect the ultrastructure of NPU cells. There are no significant different in ultrastructure between the hAM homogenate-treated NPU cells and the culture medium-treated NPU cells (ctrl). The hAM homogenate was prepared with the RH homogenizer.


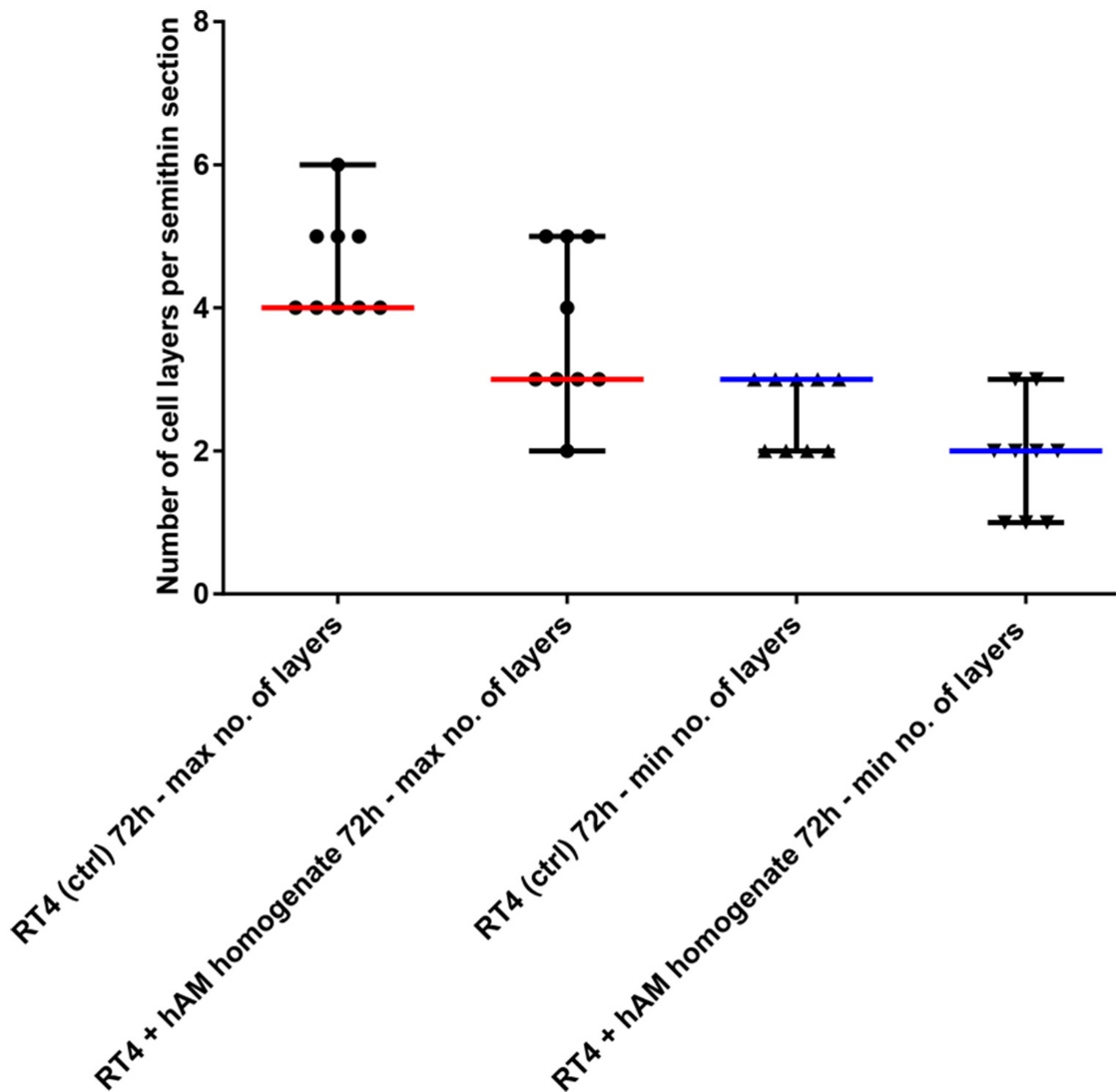


**Supplementary Figure S2.** hAM homogenate decreases the total number of RT4 cell layers after the 72-hour treatment period. The data are presented as the total number of cell layers per semi-thin sections. Data were obtained from 3 independent experiments, each performed with a different biological sample of hAM. Within each experiment, 3 technical replicates were performed.

**Supplementary Table 1.** hAM homogenate decreases proliferation of T24 and RT4 cells after 24, 48 and 72 hours of treatment. Data are shown as a percentage of proliferation rate ± SEM.

|  | **24h** | | **48h** | | **72h** | |
| --- | --- | --- | --- | --- | --- | --- |
|  | T24 (ctrl) | T24 + hAM homogenate | T24 (ctrl) | T24 + hAM homogenate | T24 (ctrl) | T24 + hAM homogenate |
| **Proliferation rate (%) ± SEM** | 27.8 ± 1.6 | 11.1 ± 0.09 | 30.2 ± 2.0 | 9.1 ± 2.0 | 18.5 ± 1.6 | 15.6 ± 2.0 |
|  | RT4 (ctrl) | RT4 + hAM homogenate | RT4 (ctrl) | RT4 + hAM homogenate | RT4 (ctrl) | RT4 + hAM homogenate |
| **Proliferation rate (%) ± SEM** | 39.9 ± 3.3 | 13.3 ± 1.0 | 43.1 ± 4.0 | 25.5 ± 1.8 | 46.6 ± 2.4 | 22.3 ± 1.4 |
